# A QbD Approach for the Formulation and Control of Triclabendazole in Uncoated Tablets: From Polymorphs to Drug Formulation

**DOI:** 10.3390/pharmaceutics16121594

**Published:** 2024-12-13

**Authors:** Lucas P. Muzi, Marina Antonio, Rubén M. Maggio

**Affiliations:** Área de Análisis de Medicamentos, Facultad de Ciencias Bioquímicas y Farmacéuticas, Universidad Nacional de Rosario and Instituto de Química Rosario (IQUIR, CONICET-UNR), Suipacha 531, Rosario S2002LRK, Argentina; muzi@iquir-conicet.gov.ar (L.P.M.); antonio@iquir-conicet.gov.ar (M.A.)

**Keywords:** polymorphism, quality by design, chemometric, dissolution prediction

## Abstract

Triclabendazole (TCB) is a well-established anthelmintic effective in treating fascioliasis, a neglected tropical disease. This study employs quality by design (QbD) to investigate the impact of TCB polymorphism and pharmacotechnical variables, from the development of immediate-release tablets to process optimization and green analysis. Critical process parameters (CPPs) and critical material attributes (CMAs), characterized by type of polymorph, composition of excipients (talc, lactose, cornstarch, and magnesium stearate), and compression force, were screened using a Plackett–Burman design (n = 24), identifying polymorphic purity and cornstarch as a CPP. To establish a mathematical model linking CPP to dissolution behaviour, a multiple linear regression (MLR) was applied to the training design (central composite design, n = 18). Simultaneously, a near-infrared spectroscopy coupled to partial least squares (NIR-PLSs) method was developed to analyze CPPs. An independent set of samples was prepared and analyzed using the NIR-PLSs model, and their dissolution profiles were also obtained. The PLSs model successfully predicted the CPPs in the new samples, yielding almost quantitative results (100 ± 3%), and MLR dissolution predictions mirrored the actual dissolution profiles (f2 = 85). In conclusion, the developed model could serve as a comprehensive tool for the development and control of pharmaceutical formulations, starting from the polymorphic composition and extending to achieve targeted dissolution outcomes.

## 1. Introduction

During the design and development of a drug formulation, a complete understanding of the properties of the active pharmaceutical ingredient (API) and the excipients becomes essential to produce safe and effective therapeutic products. Concerning formulations that employ solid APIs, this principle could be extended to the study and understanding of the physicochemical properties of the solid forms, due to their possible impact on the biopharmaceutical properties and the therapeutic performance of the formulation [1,2,3].

Currently, treatment against fascioliasis [4,5,6], a devastating neglected tropical disease, involves the use of anthelmintic drugs to eliminate the parasites and other medicines to alleviate the associated symptoms [7]. The most effective anthelmintic drug to treat this disease is triclabendazole (TCB) [6-chloro-5-(2,3-dichlorophenoxy)-2-(methylsulfanyl)-1H-benzimidazole] [8], as it being well tolerated, showing minimum adverse effects [5,7]. Despite its therapeutic effectiveness, TCB has poor water solubility (0.24 μg mL^−1^) and a variable permeability, influenced by the ingestion of food (classified alternatively as class II of the Biopharmaceutics Classification System) [9]. As a result, it is expected that the rate of dissolution is the limiting step of absorption of this API [10].

Moreover, the crystal polymorphism of TCB complicates its dissolution landscape [11,12]. Crystalline polymorphism is defined as the existence of two or more solid forms of equal chemical formula, also known as polymorphs, with different dispositions in the crystalline lattice [13,14,15]. These differences confer each polymorph’s different physicochemical properties like density, melting point, chemical and physical stability, grade and rate of dissolution, interactions with biological systems, etc. [3].

**Figure 1:**
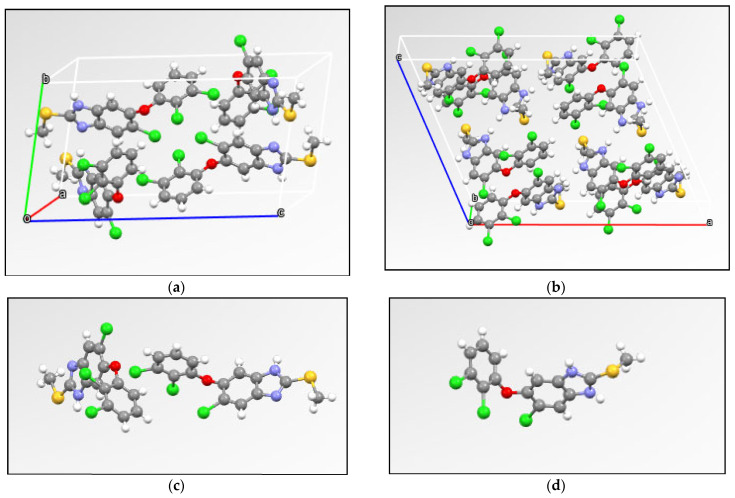
Single-crystal X-ray structures of the polymorphs Form I (CARSUL) (**a**) and Form II (CARSOF) (**b**). Conformation of asymmetric unit of Form I (**c**) and Form II (**d**).

The single-crystal X-ray structures of the TCB polymorphs (Figure 1) revealed that Form I (CARSUL) contains a triclinic symmetry cell, with a space group P-1 while Form II (CARSOF) has monoclinic cell symmetry and C 2/c space group. TCB is a rare case in which different conformers and tautomers co-exist in its polymorphic forms. Form I is composed of tautomer A in two stable conformations while the metastable Form II consists of a 1:1 mixture of tautomers A and B which appears to be unstable.

Due to the varying stabilities of polymorphs, polymorphic conversions or chemical degradation may occur during the manufacturing process and/or storage of a drug. As a consequence, unsupervised processes could result in the production of non-equivalent, useless or even unsafe products, and a possible retirement of the drug from the market [16,17]. Usually, the most stable polymorph is employed in the commercial formulation to avoid polymorphic transformations and further degradations [14,18]. However, in cases where the most stable form presents too low solubility in water, which results in low therapeutic activity, the utilization of a metastable form emerges as a suitable option [19].

The significant differences in solubility and intrinsic dissolution rate between TCB polymorphs underscore potential risks related to the bioavailability and stability of the drug. Form II exhibited a substantially higher dissolution rate than Form I, likely due to its higher energy state and lower stability [12]. Additionally, over an extended period, Form II reached a much higher concentration than Form I. Such solubility differences could impact the therapeutic efficacy and safety of formulations containing different polymorphs, particularly if Form II converts to the more stable Form I over time, potentially reducing its solubility and thus its bioavailability in the body. Therefore, careful consideration of polymorph stability and dissolution behaviour is essential in formulation development and quality control.

Pharmaceutical development aims for the design and production of a quality product to consistently deliver an intended performance [20]. Quality by design (QbD) consists of a systematic approach to the development and continuous improvement of a productive process, with an emphasis on the integral comprehension of the desired product and optimizing the manufacturing process. Its principal objective is to develop robust processes and products that maintain the desired attributes of quality along with a good product shelf life, resulting in a reduction in variability and ensuring drug product equivalence. To fully understand the sources of variability, QbD relies on solid scientific knowledge (about the process, raw materials, and products), risk management, design of experiments (DoEs) [21], and process analytical technologies (PATs) [20]. Empirical models obtained during DoE steps and production of formulations are very useful in the determination of the critical process parameters (CPPs) and critical material attributes (CMAs) that make a drug safe and effective [22]. PATs supervise and directly or indirectly monitor the critical quality attributes (CQAs). The implementation of a chemometrics-based PAT results in an overall increase in the efficacy of the process and assurance of the quality, and it reduces the time of analysis and solvent consuming, minimizing the impact on the environment [23,24].

The major CQA of solid and semi-solid pharmaceutical forms is drug dissolution since it is often the limiting step among the dossing and bioavailability [25,26]. For this reason, dissolution tests have become essential in vitro experiments suited for determining batch-to-batch reproducibility [27]. The analysis of dissolution profiles is used in specific cases to test the performance of new formulations, ensuring drug equivalence after minor production changes and even predicting the in vivo bioavailability of drug products [28,29].

In this work, the implementation of a QbD strategy to analyze how TCB polymorphism and others CPPs influence the dissolution of tablets by establishing mathematical models and mapping the design space (DS) of the dissolution was proposed. In parallel, a PAT-based on a partial least squares (PLSs) model will be developed to quantitate the TCB polymorphs and other CPPs using near-infrared spectroscopy (NIR). The aim of this work was to estimate whether the tablets meet the conditions to remain within the defined DS. To the best of our knowledge, no existing studies combine the investigation of polymorphism and other CPPs with the development of PAT and the application of both to evaluate the dissolution of intact tablets.

## 2. Materials and Methods

### 2.1. Instrumentation

NIR spectra were acquired in a spectrometer NIRS DS2500 FOSS (FOSS, Hillerod, Denmark). Measurements were obtained at room temperature, using reflectance mode. All determinations were carried out in a slurry cell equipped with an adaptor for tablets. All the tablets were measured in the spectral range of 800–2500 nm.

Dissolution profiles were obtained in a Hanson SR8-Plus dissolution station (Hanson Research, Chatsworth, LA, USA), configured as USP apparatus II (paddles) [30] with a rotation rate of 100 rpm. A dissolution medium containing HCl with ethanol 96° (EtOH) was prepared by adding a solution of HCl (pH 1.2, without KCl) to 200, 300, or 400 mL of EtOH to reach 1000 mL in a volumetric flask.

The amount of TCB dissolved was determined by spectroscopic measurements at 305 nm, using a Shimadzu UV-1601PC spectrophotometer (Shimadzu Corp., Kyoto, Japan). Determinations were performed in a quartz cell (10 mm optical path length) against a blank dissolution medium.

The particle size of each sample was standardized via sieving, employing a Zonytest EJR 2000 vertical vibrational sieving tower (Rey & Ronzoni, Buenos Aires, Argentina), operating at 1200 rpm. In all cases, the fractions 100–140 mesh were collected.

The physical mixtures of solids were homogenized at 30 rpm for 15 min. A Z-mixer moved using a rotary platform with an AT-15D electronic control of speed (Precytec, Buenos Aires, Argentina) was employed for this purpose.

### 2.2. Chemicals

The TCB used was a kind gift from Farmavet Laboratories (Farmavet, Rosario, Argentina). The excipients used (talc, lactose, cornstarch, and magnesium stearate) were of pharmaceutical grade and purchased from Saporiti Laboratories (Parafarm, Buenos Aires, Argentina). All other reagents and solvents were of analytical grade and were used as received.

### 2.3. The Obtention of TCB Polymorphs

Polymorph I (FI) of TCB was obtained by mixing 1 g of commercial TCB with 35 mL of MeOH. The mix was settled at room temperature, and the crystals were obtained via slow evaporation over 4–6 days [12].

Polymorph II (FII) of TCB was obtained from an oversaturated solution of acetonitrile heated up to 82 °C. The reflux was maintained for 15 min, and then the solution was hot-filtered and left to cool down to room temperature. The crystals were obtained via multiple filtrations and dried in a vacuum oven at 60 °C. The first-filtered crystals were discarded to improve polymorphic purity [12]. Solid phases were maintained in a desiccator at room temperature and protected from daylight. The polymorphic identities of both forms were confirmed via X-ray powder diffraction (XRPD) and differential scanning calorimetry (DSC), which have been similarly performed by other studies (Figure S1).

### 2.4. Tablets Preparation

Polymorphs and excipients were individually sieved, and the fractions corresponding to 100–140 mesh were collected. For every different tablet composition, the polymorphs and excipients were weighted and mechanically mixed for 15 min at 30 rpm in a Z-mixer and then compressed to tablets. The order of the powder addition was as follows: first lactose and API, followed by a homogenization step. Then, cornstarch and Talc was added, and the mixture was homogenization again. Finally, magnesium stearate was added. Each average tablet was approximately 310 mg (250 mg of TCB, 20 mg of talc, 15 mg of lactose, 15 mg of cornstarch, and 10 mg of stearate magnesium).

### 2.5. Chemometrics and Software

Exploratory analysis of data, statistics, and graphs was performed using Origin 8.0 (OriginLab Co., Northampton, MA, USA) and Microsoft Office Excel 2016 (Microsoft, Redmond, WA, USA). Partial least squares (PLSs) [31] calculations were carried out using the MVC1 Toolbox run in Matlab R2010a (Mathworks, Natick, MA, USA). Experimental designs and calculations were computed using Design-Expert 7.0 (Stat-Ease, Minneapolis, MN, USA).

## 3. Results and Discussion

The major issue of TCB that could compromise its therapeutic efficacy is its poor water solubility, resulting in a low grade of dissolution and thus a low grade of absorption [10,32,33]. Additionally, polymorphs hold significant differences in solubility and intrinsic dissolution rate as previously stated. Form II exhibited a substantially higher intrinsically dissolution rate and solubility over an extended period (21 h) compared to Form I. This is due to Form II’s higher energy state [12]. Such solubility differences could impact the therapeutic efficacy and safety of formulations containing different polymorphs, and a careful consideration of polymorph stability and dissolution behaviour is essential in formulation development. The general QbD working strategy here implemented starts with the definition of potential CPPs and CQAs for the utilized tablets. This is established through a combination of prior scientific knowledge and a risk analysis of relevant factors. In our particular case, the quality attribute that is intended to be ensured through QbD is the pharmaceutical dissolution [34] given the crystalline polymorphism of the drug and its classification as class II in the Biopharmaceutics Classification System.

A statistical analysis was conducted to examine CPPs that exert a significant influence on the dissolution performance. The identification of such CPPs responsible for dissolution behaviour was carried out using the design of experiment tools. A Plackett–Burman design (PBD, n = 24), where the composition of excipients (talc, lactose, cornstarch, and magnesium stearate), polymorphic content, and force of compression (Table 1) were studied as model factors. 

**Figure 2:**
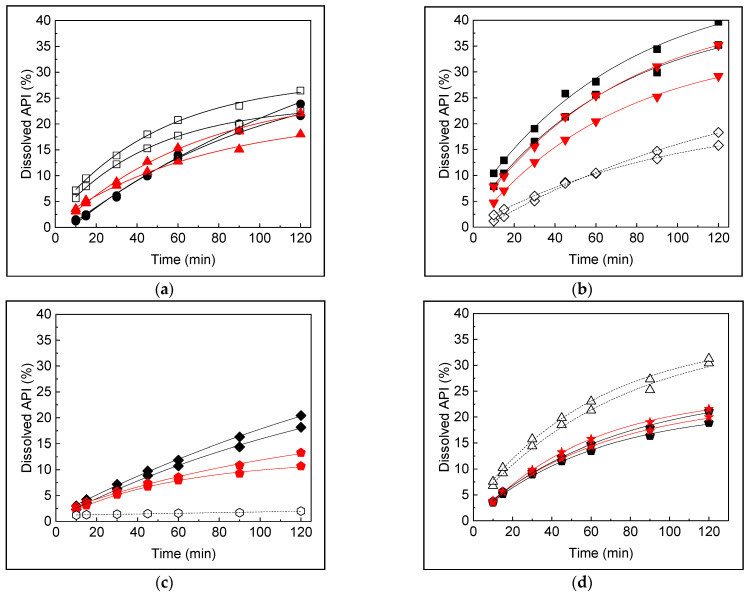
Dissolution profiles of samples employed in the Plackett–Burman design. (**a**) Samples 1 (●), 2 (▲), and 3 (□); (**b**) samples 4 (■), 5 (◇), and 6 (▼); (**c**) samples 7 (◆), 8 (⬟), and 9 (⬡); and (**d**) samples 10 (△), 11 (⬟), and 12 (★).

The dissolution profiles obtained from PBD (Figure 2) were analyzed over time using a stepwise ANOVA procedure; thus, the model retains only the factors that reached the minimum confidence level. The significance of the factors was determined with a 95% confidence, and the results are presented in Table 2.

Cornstarch and magnesium stearate were found to be significant for dissolution up to 15 min, whereas cornstarch and the polymorphic type were significant until the dissolution process reaches the plateau. Compression force would be expected as a CPP as well; however, this variable presented no significance in the range studied. Magnesium stearate was not considered for further analysis due to its marginal impact, which was observed only in the initial stages when dissolution rates were still very low. Lastly, the amount of magnesium stearate used in the formulation should be fixed to ensure the flowability of the mixture through the hopper; therefore, it cannot be considered for the optimization of the model [35].

### 3.1. Development of an Empirical Model for the Study of Dissolution Profiles

With the aim of obtaining an empirical model for the prediction of dissolution profiles, quantitative relationships between CPPs and dissolution behaviour were studied by response surface methodology. A central composite design (CCD) of two factors at five levels (n = 18) was carried out, where the variables analyzed were FI content (expressed as % *w*/*w*) and amount of cornstarch per tablet (Table 3); the rest of the components were maintained as established in Section 2.4.

**Figure 3:**
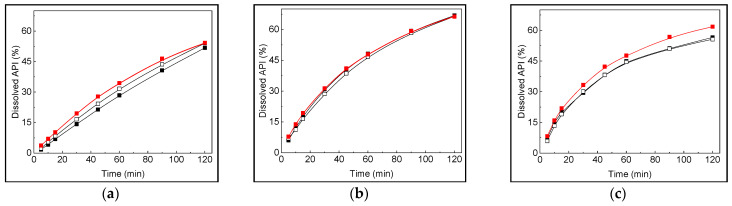
Dissolution profiles of test (■ and □) and predicted (■). Tablets with 12.5 mg of cornstarch and 15% of FI (**a**), 24 mg of cornstarch and 50% of FI (**b**), and 36 mg of cornstarch and 87% of FI (**c**).

Starting from the dissolution results (Figure 3), a separate prediction model was obtained for the dissolution at every time profile (Figures S3 and S4). Table S1 summarize the stepwise ANOVA results of the analysis of the quadratic model with first-level interactions. The models obtained were significant at all times.

Both CPPs (FI content and amount of cornstarch) results were significant at all dissolution times. Cornstarch had a quadratic relationship with dissolution behaviour while the FI content had only a linear correlation. The equations are shown in Table 4. To evaluate the prediction models, a validation set of tablets varying both CPPs (n = 10) was tested. The dissolution profiles of three samples with low, middle, and high levels of CPPs are depicted in Figure 3 (all samples dissolution profiles are shown in Figure S5). The comparability of the actual vs. predicted profile could be observed at first glance.

Further mathematical analysis to compare actual and predicted profiles was carried out by calculating the parameter f_2_ [36]. All values obtained for f_2_ were in the range of 80–100 (Table S2), indicating full equivalence of actual and predicted dissolution profiles. The relevance of these results lies in the possibility of defining the DS where CPPs bring an acceptable value of CQA. The assurance of CQA inside a pre-established confidence interval would guarantee the quality of the product, which could be achieved by controlling the values of the CPPs using PAT.

### 3.2. Definition of Design Space

**Figure 4:**
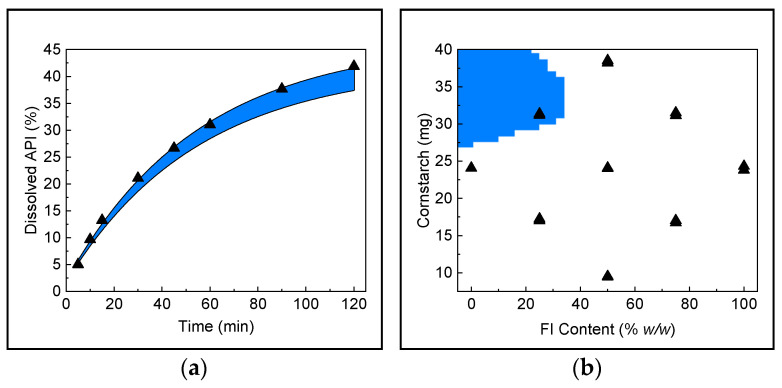
Maximum dissolution profile (▲) and the dissolution region with an f_2_ > 85 (blue) (**a**). CPP’s region that yields a f_2_ > 85 dissolution performance (**b**).

The construction of a DS is essential to delimit the region where the pharmaceutical product can be obtained with the assurance that it will meet the quality standards established for the CQA. For this purpose, the region was delimited based on the values of both CPPs, FI content, and amount of cornstarch that generate dissolution profiles with an f_2_ factor of no less than 85 when concerning the maximum dissolution. In the first instance, a calculation is made of the minimum dissolution profile that provides the limit value of f_2_ (Figure 4a), and subsequently, the region of factors that provide profiles in the interval between the maximum profile and the profile f_2_ = 85 was calculated using Derringer’s desirability function (Figure 4b).

Based on the results obtained from DS, combinations of CPPs that provide an acceptable dissolution profile without implying a change in tablet quality can be established. The content of FI should not exceed 20%, and the amount of cornstarch per tablet cannot be less than 30 mg. However, particular combinations that are very close to the boundary can be directly compared with the previously obtained or even be analyzed using the empirical model to calculate their respective dissolution profiles.

#### 3.2.1. Quantification of CPP Using a NIR-PLS Method

**Figure 5:**
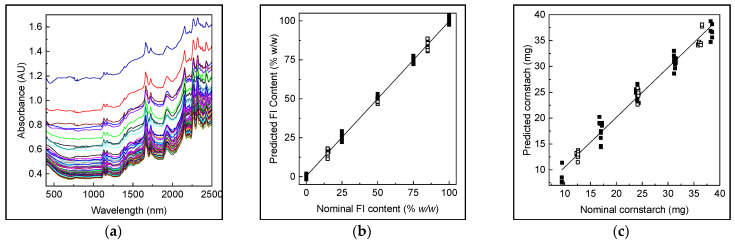
Raw NIR data for the calibration and validation set (**a**). Actual vs. predicted values of FI content in entire tablets for the calibration (■) and validation set (□) (**b**). Actual vs. predicted values of cornstarch in tablets for the calibration (■) and validation set (□) (**c**).

Once established, the boundaries of DS was necessary to develop the analytical method for determining and controlling CPPs responsible for dissolution behaviour to assure the quality of the final products, and, in this way, assure the batch-to-batch equivalence in production [37]. The direct analysis of tablets, where the mixture of excipients interferes with the detection and quantification of every component and also API polymorphs, makes it necessary to associate the NIR with chemometrics to extract the maximum information of the analytes of interest [38]. NIR proved to be an accurate and precise tool for quantifying polymorphs in drug substances and products [39,40,41]. Specifically, in our previous study, NIR-PLS was suitable for determining both TCB polymorphs in different TCB forms and tablets [12], while MIR spectroscopy was prone to interference caused by the polymorphs. For the training set (n = 18, Section 3.1) entire tablets were examined three-fold, yielding a total of 54 spectra (Figure 5a), similar to the validation set (n = 10), obtaining 30 spectra. The training and validation data were pre-treated with multiplicative scattering correction (MSC) and mean centred (MC), and the spectral region was optimized via interval PLS [42] to enhance the performance of calibration, determining the interval 925–2500 nm as the optimal one for both CPPs.

Evaluation of the number of PLS factors was carried out by cross-validation procedures (leave-one-out), where the number of factors was selected according to a minimum value of PRESSs (prediction error sum of squares). Table 5 summarizes the calibration parameters. As seen in Figure 5b,c, the actual vs. predicted value curves for both CPPs showed excellent linear regression. The values of R^2^ were over 0.95 for both models, indicating a good fit of the experimental data to the calibration curve.

The recovery percentage was not statistically different from 100%, with REP values close to 5% (5.14 and 5.17%, for the content of FI and cornstarch, respectively), which correlated with low RMSEP values [2.52 (% *w*/*w* of FI) and 1.25 (mg), for FI content and cornstarch, respectively]. Table 5 summarizes the validation results and performance indicators of each model. The relevance of these results lies in the implications of ensuring the correct API’s grade of dissolution from the control of the CPP. This could turn into a rapid, non-invasive, online PAT to apply in the routine quality control of the pharmaceutical industry for tablets of TCB.

#### 3.2.2. Dissolution Prediction of Actual Samples

**Figure 6:**
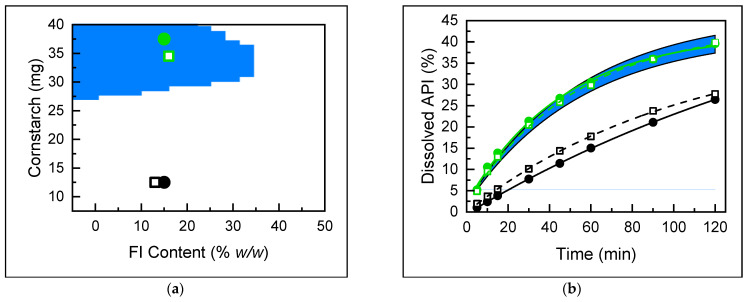
Actual (●) and estimated (□) CPPs values of T1 (green) and T2 (black) overlapped with the DS (blue region) (**a**). Experimental (-●-) and predicted (--□--) dissolution profiles of T1 and T2 overlapped with the DS dissolution projection (blue region) (**b**).

In order to demonstrate the overall applicability of the methodology into the QbD paradigm, the prediction of dissolution profiles of intact tablets and the determination of their suitability were carried out. Two different samples (T1 and T2) were analyzed, and their CPPs were determined by NIR-PLS (Figure 6a). The values of CPPs (polymorph and cornstarch contents) were used as input for a multiple linear regression (MLR) model to evaluate their dissolution. The predicted dissolution curves obtained were compared to their actual curves and overlapped with the DS dissolution projection (Figure 6b).

As can be seen, tablet T1 met the polymorphic and cornstarch contents (15% and 37.5 mg, respectively) in the DS to obtain an acceptable dissolution. On the other hand, T2 (15% *w*/*w* FI and 12.5 mg cornstarch) does not achieve the level of dissolution to be considered suitable for both MLR-predicted and actual dissolution profiles. In this way, we demonstrated a full coincidence between the achieved results via actual and estimated dissolution evaluation.

## 4. Conclusions

A quality by design (QbD) approach to investigate the impact of crystal polymorphism of TCB and other pharmacotechnical variables for the development of immediate-release tablets was carried out. The influences of CPPs and CMAs, polymorphs, composition of excipients (talc, lactose, cornstarch and magnesium stearate), and compression force, on the CQA dissolution were screened using a PBD (n = 24), where FI and cornstarch contents emerged as relevant CPPs. The empirical model was established using MLR through a CCD (n = 18) to analyze the statistical significance of CPPs. An independent set of samples was prepared, and their dissolution profiles were also obtained (n = 10). Results from the models mirrored the actual dissolution profiles obtained from each tablet (average f_2_ = 85). Simultaneously, two NIR-PLS models were developed and validated to determine the FI and cornstarch contents in entire tablets showing accuracy and prediction during cross-validation. The PLS models successfully predicted both CPPs in new samples, yielding almost quantitative results (100 ± 3%). For the sake of demonstrating the full applicability of the model, two TCB tablets were analyzed using the NIR-PLS model, and their CPPs were determined and used to predict dissolution profiles. The predicted dissolution profiles showed no differences from the actual ones, allowing us to evaluate their suitability when compared to the DS dissolution projection.

In conclusion, the developed dissolution model, in combination with NIR-PLS, demonstrates potential as a comprehensive green QbD tool for controlling and predicting the dissolution behaviour of TCB tablets by analyzing their polymorphism and other CPPs. However, its full implementation will require validation on a larger scale and within an industrial environment.

## Figures and Tables

**Table 1:** Plackett–Burman design for tablets compositions.

Sample	Form of TCB	Compression Force (kN)	Talc (mg)	Lactose (mg)	Cornstarch (mg)	Magnesium Stearate (mg)
1, 1′	II	12	39.5	29.3	14.3	19.4
2, 2′	I	12	19.2	14.8	14.4	9.5
3, 3′	I	16	19.1	28.9	29.0	11.2
4, 4′	II	12	39.0	29.1	29.0	9.6
5, 5′	I	12	19.5	29.2	14.3	19.2
6, 6′	II	16	19.4	28.8	28.9	19.1
7, 7′	II	16	39.3	14.4	14.6	10.0
8, 8′	I	16	38.9	29.0	14.7	9.7
9, 9′	II	16	19.2	14.6	14.9	19.1
10, 10′	II	12	19.5	14.8	29.4	9.7
11, 11′	I	12	38.8	14.5	29.9	19.3
12, 12′	I	16	38.8	14.7	28.8	19.2

**Table 2:** Summary of Plackett–Burman design results using ANOVA.

Time (min)	Model (P)	Factor 1	(P)	Factor 2	(P)	SD (%)	R2
5	0.0004	Cornstarch	0.0002	Magnesium stearate	0.005	0.43	0.862
10	0.001		0.0006		0.01	1.2	0.817
15	0.0006		0.0003		0.01	1.4	0.847
30	0.003		0.002	Type of polymorph	0.08	2.3	0.772
45	0.004		0.003		0.05	2.9	0.755
60	0.003		0.003		0.03	3.2	0.770
90	0.002		0.004		0.009	3.4	0.790
120	0.003	Type of polymorph	0.005	Cornstarch	0.006	3.8	0.793

**Table 3:** Central composite design for tablet compositions.

Sample	Polymorphic Purity (% *w*/*w*)	Cornstarch (mg)	Sample	Polymorphic Purity (% *w*/*w*)	Cornstarch (mg)
1	24.9	31.2	1’	25.0	31.3
2	50.0	38.2	2’	50.0	38.5
3	50.0	9.5	3’	50.1	9.5
4	75.0	17.0	4’	75.0	16.7
5	49.9	24.0	5’	49.9	24.1
6	24.9	17.2	6’	25.0	17.0
7	75.0	31.1	7’	75.0	31.5
8	100.0	23.8	8’	100.0	24.3
9	0.0	24.0	9’	0.0	24.1

**Table 4:** Equations of the response surfaces for each time.

Time (min)	Equation	Equations
5	$D=-2.32-0.01\times A+0.43\times B-0.006\times B^{2}$	(1)
10	$D=-2.68-0.02\times A+0.63\times B-0.008\times B^{2}$	(2)
15	$D=-3.45-0.03\times A+0.88\times B-0.011\times B^{2}$	(3)
30	$D=-2.38-0.055\times A+1.27\times B-0.017\times B^{2}$	(4)
45	$D=-0.255-0.068\times A+1.50\times B-0.021\times B^{2}$	(5)
60	$D=+1.63-0.086\times A+1.68\times B-0.024\times B^{2}$	(6)
90	$D=+6.48-0.11\times A+1.84\times B-0.027\times B^{2}$	(7)
120	$D=+9.78-0.13\times A+1.94\times B-0.029\times B^{2}$	(8)

D = dissolved TCB (%), A = FI content (% *w*/*w*), and B = cornstarch (mg).

**Table 5:** Calibration summary, figures of merit, and validation results of PLS regression/CPPs determination.

Parameter	FI Content	Cornstarch
	Calibration summary	
Calibration samples	54	
Concentration levels	5	
Latent variables	6	
Spectral range (nm)	925–2500	
Calibration range	0–100 (% *w*/*w*)	10–40 (mg)
Pre-treatment	MC, MSC ^a^	
	Figures of merit	
R^2 b^	0.9974	0.9947
Sensibility	0.0060	0.0148
Analytical sensibility (γ)	7.56	2.024
REP (%) ^c^	5.14	5.17
RMSE ^d^	2.52 (% *w*/*w*)	1.25 (mg)
LOQ ^e^	4.89 (% *w*/*w*)	6.53 (mg)
	Results and statistics of validation	
Validation samples	30	30
Recovery	103.65	99.93
RSD ^f^ (%)	11.14	5.16

^a^: multiplicative scattering correction; ^b^: coefficient of determination (R²); ^c^: relative error in prediction (REP); ^d^: root mean square error (RMSE); ^e^: limit of quantification (LOQ); and ^f^: relative standard deviation (RSD).

## Data Availability

The raw data will be available upon request from the authors.

## Supplementary Materials

The following supporting information can be downloaded at the following website: https://www.mdpi.com/article/10.3390/pharmaceutics16121594/s1. Figure S1: Diffractograms and thermograms of FI and FII of TCB; Figure S2: Dissolution profiles of samples employed in the modelling; Figure S3: Response surfaces of dissolution of TCB tablets at first times; Figure S4: Response surfaces of dissolution of TCB tablets at ending times; Figure S5: Actual and predicted dissolution profiles of test TCB tablets; Table S1: Statistical analysis of central composite design; Table S2: Values of f_1_ and f_2_ for test TCB tablets.
