# Supplementary material for: A QbD Approach for the Formulation and Control of Triclabendazole in Uncoated Tablets: From Polymorphs to Drug Formulation
Source: Pharmaceutics. 2024 Dec 13;16(12):1594. doi: 10.3390/pharmaceutics16121594 (PMC11679758; doi:10.3390/pharmaceutics16121594)
Supplement: Supplementary file 1 [file pharmaceutics-16-01594-s001.zip › pharmaceutics-3341498-supplementary.pdf]

pharmaceutics

MDPI

Supplementary Materials

A QbD Approach for the Formulation and Control of Triclabendazole in Uncoated Tablets: From Polymorphs to Drug Formulation

Lucas P. Muzi, Marina Antonio and Rubén M. Maggio

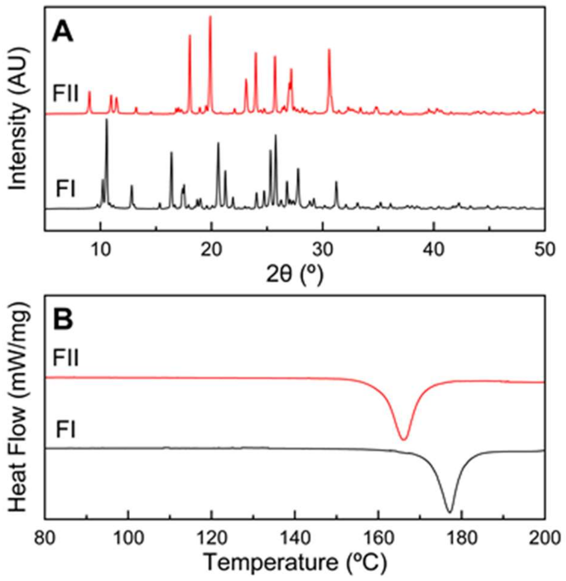

Figure S1. Diffractograms (**A**) and thermograms (**B**) of FI and FII of TCB.

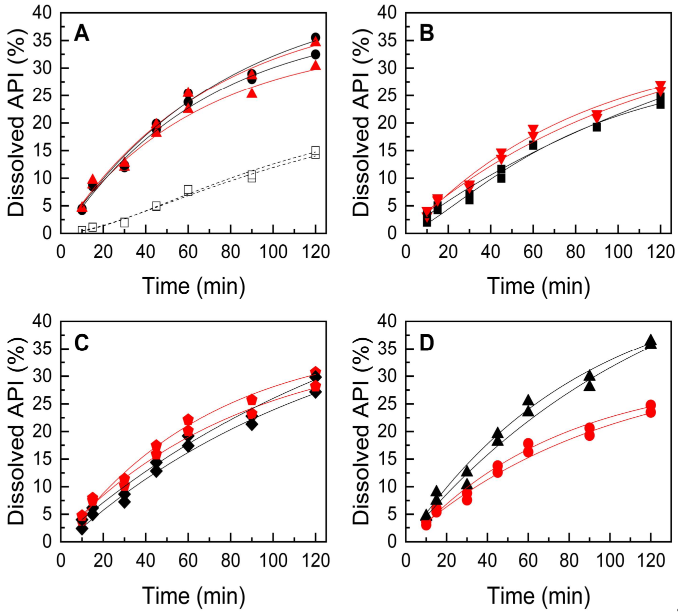

Figure S2. Dissolution profiles of samples employed in the modeling. (**A**) Samples 1(●), 2(▲) and 3(□); (**B**) samples 4(■) and 5(▼); (**C**) samples 6(◆) and 7(⬟); (**D**) samples 8(●) and 9(▲).

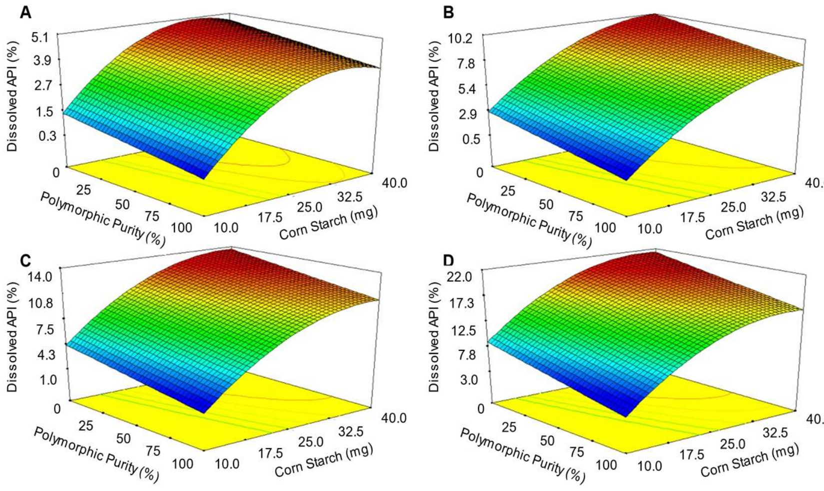

Figure S3. Response surfaces of dissolution of TCB tablets at first times:5 min (**A**), 10 min (**B**), 15 min (**C**) and 30 min (**D**).

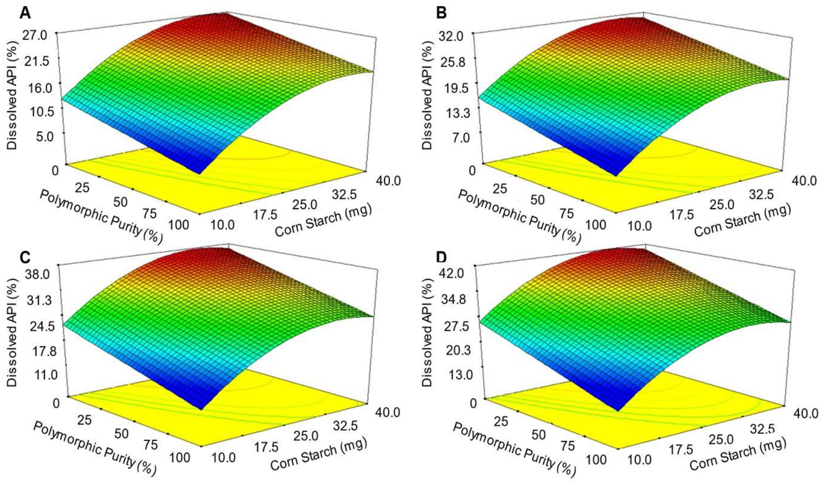

Figure S4. Figure S4: Response surfaces of dissolution of TCB tablets at ending times; 45 min (**A**), 60 min (**B**), 90 min (**C**) and 120 min (**D**).

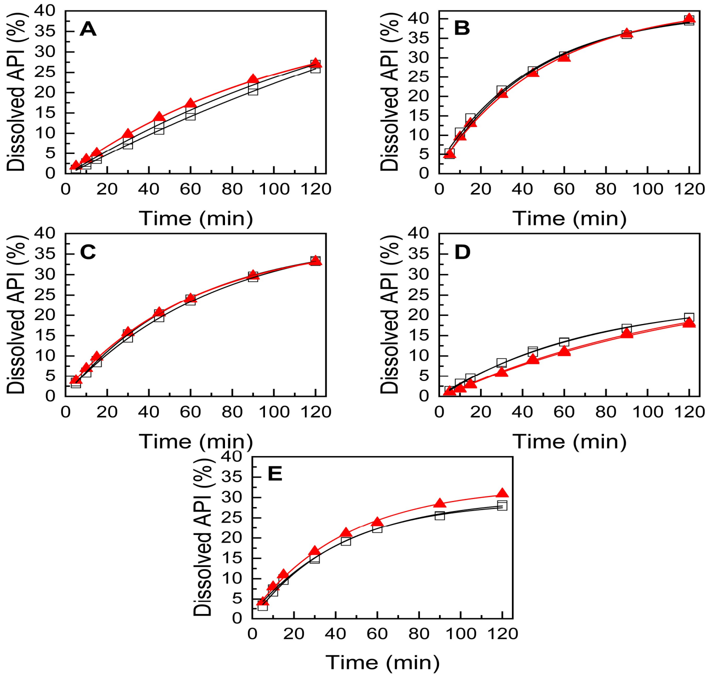

Figure S5. Actual (black) and predicted (red) dissolution profiles of test TCB tablets. Tablets with: 12.5 mg of corn starch and 15% of FI (**A**), 36 mg of corn starch and 15% of FI (**B**), 24 mg of corn starch and 50% of FI (**C**), 12.5 mg of corn starch and 85% of FI (**D**), 36 mg of corn starch and 85% of FI (**E**).

Table S1. Statistical analysis of Central composite design.

Time (min)	5	10	15	30	45	60	90	120
Model (*P*)	<0.0001	<0.0001	<0.0001	<0.0001	<0.0001	<0.0001	<0.0001	0.0015
Polymorphic purity (*P*)	0.0519	0.0005	0.0008	<0.0001	<0.0001	<0.0001	<0.0001	<0.0001
Corn Starch (*P*)	<0.0001	<0.0001	<0.0001	<0.0001	<0.0001	<0.0001	<0.0001	<0.0001
Corn Starch^2 (*P*)	0.0027	0.0010	0.0007	<0.0001	0.0006	0.0006	0.0011	0.0008
SD (%)	0.58	0.62	0.86	1.07	1.55	1.73	2.19	2.28
R^2	0.845	0.949	0.942	0.956	0.928	0.928	0.903	0.905

Table S2. Values of f_1 and f_2 for test TCB tablets.

Sample	f_1	f_2	Sample	f_1	f_2
A	16.7	80.0	A′	7.9	91.1
B	1.91	97.5	B′	3.6	93.2
C	2.2	97.43	C′	4.9	92.2
D	22.0	83.1	D′	18.9	85.5
E	10.4	82.5	E′	8.7	84.4

$f_2 = 50 \times \log\{[1 + (1/n) \times \sum_{t=1}^{n}(R_t - T_t)^2]^{-0.5} \times 100\}$, where R_t are the reference measurements of dissolved API at different times and T_t are the test measurements.
